# ESIPT-active 8-hydroxyquinoline chemosensor for highly selective detection of diethyl chlorophosphate and molecular logic gate applications

**DOI:** 10.1039/d6ra01529h

**Published:** 2026-05-11

**Authors:** Aastha Palta, Gulshan Kumar, Kamaldeep Paul, Vijay Luxami

**Affiliations:** a University Centre for Research and Development, Chandigarh University Mohali-140413 India; b Department of Chemistry, Banasthali University Banasthali Newai 304022 Rajasthan India; c Department of Chemistry and Biochemistry, Thapar Institute of Engineering and Technology Patiala-147004 India vluxami@thapar.edu

## Abstract

An ESIPT-active 8-hydroxyquinoline-based fluorescent chemosensor, HQHBI, has been synthesized for the sensitive detection of the nerve-agent simulant diethyl chlorophosphate (DCP). The probe exhibits a low detection limit of 1.5 × 10^−7^ M and demonstrates excellent selectivity in the presence of a wide range of competing anions. Photophysical studies in different solvents reveal the involvement of ESIPT and ICT processes, while pH titrations confirm its operational stability under physiological and environmental conditions. The sensing mechanism is further validated through time-resolved fluorescence and NMR titration studies, indicating selective interaction *via* the phenolic –OH group. Additionally, DFT and TD-DFT calculations support the experimental findings by elucidating key electronic transitions responsible for the sensing response. Notably, HQHBI also enables molecular logic gate operations, highlighting its potential in chemical information processing.

## Abstract 2

An ESIPT-active 8-hydroxyquinoline-based fluorescent chemosensor, HQHBI, has been synthesized for the sensitive detection of the nerve-agent simulant diethyl chlorophosphate (DCP).

## Graphical abstract

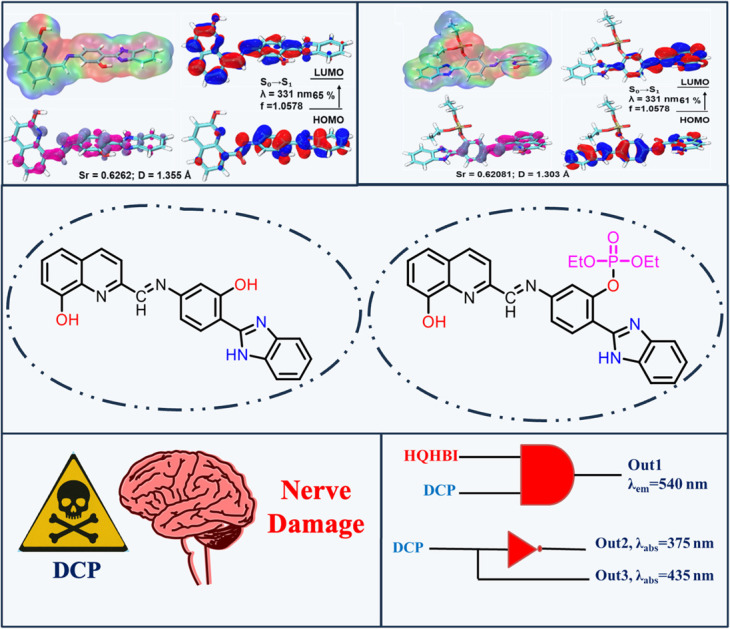

## 1. Introduction

Selective, rapid, and sensitive detection of organophosphates (OPs) has attracted strong worldwide interest because they serve as a powerful agent in chemical warfare and are also widely used as agro pesticides. Their high acute toxicity and widespread use in agricultural areas make OPs one of the greatest threats not only to the environment but also to human health, civic security, and defense.^1,2^ Hence, there is an urgent need to develop swift, dependable, and extremely sensitive methods for the detection of OP tracking to safeguard human health, monitor environmental pollution, and strengthen responses to chemical threats. Conventional methods for OP detection, such as gas chromatography, high-performance liquid chromatography, and mass spectrometry, are highly accurate; however, their application is limited by factors such as high cost, the requirement for sophisticated instrumentation, extensive sample pre-treatment, and their limited suitability for real-time or on-site detection.^3–5^ These limitations have driven researchers to explore alternative detection techniques that are rapid, selective, and highly sensitive, with the potential for on-site applications. Among the less hazardous model compounds, diethyl chlorophosphate (DCP) is widely employed as a simulant for organophosphate nerve agents.^6–11^ DCP is known to have the reactive phosphoryl moiety of these highly toxic agents, but it is less dangerous, which will make it a good candidate in the study of recognition mechanisms and validation of sensor performance. Consequently, creation of fluorescent sensors of DCP has become a major research area.

Fluorescent chemosensors have received particular attention during the last 10 years due to their exceptional sensitivity, simplicity, quickness and the visual message or readout, which is commonly seen by the naked eye or low-cost portable instruments. Among them, chemosensors working on the principle of the Excited-States Intramolecular Proton Transfer (ESIPT) have demonstrated the most potential.^12–15^ The photophysical process known as ESIPT is where an intramolecular proton is relayed to an acceptor (*e.g.* an imine or heteroatom) in the excited state of a donor (probability most commonly a hydroxyl group). This transfer leads to the generation of tautomeric excited states, often producing dual emission bands or large Stokes shifts, properties that significantly improve the sensitivity and selectivity of detection.^16–20^ Moreover, ESIPT-based sensors are usually characterized by high photostability and environmental responsivity, which means that they are especially appealing to the application regarding chemical sensing and bioimaging. The photophysical responses can be enhanced by the introduction of two independent ESIPT centers in one molecular scaffold to obtain stronger emission signals with increased selectivity and multiple output channels towards ratiometric sensing.^21–26^ These dual systems offer distinct advantages, including minimized background interference, improved signal-to-noise ratios, and the potential for quantitative analysis.

The 8-hydroxyquinoline (8-HQ) has become a building block that is highly flexible and useful in the development of ESIPT sensors.^27–29^ The 8-HQ has a phenolic –OH functional site on the side of a nitrogen heteroatom and this further allows it to be strongly hydrogen bonded and forms the most perfect donor–acceptor system of ESIPT. The structural tunability of 8-HQ permits facile functionalization at numerous spots which enables the addition of more donor/acceptor groups, conjugated π-systems and solubilizing substituents to adjust photophysical and recognition characteristics. The capacity of 8-HQ to stabilize the interaction with phosphoryl oxygens is of the greatest value in terms of OP detection.^30,31^

Over the past years, it has become common to find researchers integrating the ESIPT properties of 8-HQ with rational functionalization strategies to engineer the design of double ESIPT-active systems that can offer strong and selective responses to DCP. It was found that the functionalization of 8-HQ with other hydrogen donor–acceptor motifs could allow the existence of multiple ESIPT different pathways, which leads to stronger fluorescence turn-on responses in the presence of DCP. The action of this system is that when oxygen of DCP is phosphorylated with high affinity to the functional groups of the sensor, it alters the intramolecular hydrogen bonding and opens the ESIPT channels, resulting in a demonstrable fluorescence emission. This not only enable the detection of highly sensitive levels with low concentrations of OP but also real-time measurements of OP exposure in mild experimental conditions.

The present work depicts the design and synthesis of 8-hydroxyquinoline (8-HQ) chemosensor that has been designed possessing dual ESIPT sites specifically to detect DCP. The sensor was strategically engineered to harness the intrinsic ESIPT capability of the 8-HQ core while incorporating an additional hydrogen-bond donor–acceptor motif through structural modification. The absorption band of HQHBI showed a strong band at 370 nm, attributed to the π–π* transition of the conjugated system. Upon excitation at this wavelength, the sensor emits a bright fluorescence emission at 535 nm with a Stokes shift of 165 nm. This large shift is highly advantageous for minimizing self-absorption and background interference, thereby improving detection accuracy. Beyond sensitivity and selectivity, the practical applicability of ESIPT-based 8-HQ sensors lies in their rapid response times, operational simplicity, and ability to function under near-physiological conditions. Such features make them not only suitable for laboratory-based studies but also highly relevant for field applications, such as environmental monitoring of pesticide residues, rapid screening of contaminated water sources, and deployment in defence-related scenarios for nerve agent detection. The combination of strong photostability, large Stokes shifts, and high selectivity ensures that these probes can perform reliably even in complex and interfering environments.

## 2. Experimental section

### 2.1. Materials and characterizations

All reactions were done without further purification using commercially available reagents (Sigma-Aldrich, Spectrochem Pvt. Ltd, India). Reaction progress was followed by TLC on silica gel HF-254 using UV light (365 nm) and column chromatography (SiO_2_, 60–120 mesh) was used to purify products. ^1^H and ^13^C NMR spectra were obtained on a Bruker 400 MHz spectrometer and chemical shifts (*δ*) were referenced to TMS.

A photophysical solution was made by preparing a stock solution of HQHBI (10^−3^ M) in acetonitrile (CH_3_CN) and diluted. The spectra of UV-vis absorption were registered at a SHIMADZU-2600 spectrophotometer (1 cm quartz cuvette), and the spectra of fluorescence were obtained at a Varian Cary Eclipse spectrophotometer (excitation/emission slit widths 5 nm). The data were exported in ACS files and converted to Excel^tm^.

Calculations were obtained at the B3LYP/6-31G* level of DFT calculations with Gaussian 16. Tetrabutylammonium salts of stock solutions (10^−1^ M) of different anions F^−^, Cl^−^, Br^−^, I^−^, HSO_4_^−^, P_2_O_7_^4−^, ClO^−^, H_2_PO_4_^−^, CN^−^, SCN^−^, NO_3_^−^, AcO^−^, DCP, triethylphosphate (TEP), or tributylphosphate (TBP) were made in deionized water and diluted.

#### 2.1.1. Computational details

The synthesized compound was carefully optimized using the Becke three-parameter Lee–Yang–Parr (B3LYP) exchange functional with the 6-311g(d) basis set. The optimized structure was validated by confirming the absence of imaginary frequencies, ensuring that the geometry corresponded to a true local minimum. Interestingly, the synthesized compound exhibits notable molecular flexibility, which allows for the possibility of multiple isomeric conformations. During the conformational search, we have observed four conformers as C1, C2, C3, and C4, among these C3 and C4 forms emerged as the most stable and was therefore selected for further investigation. Its electronic spectra were calculated using TDDFT/B3LYP/6-311g(d). We have also explored the optimization and electronic spectra at different functional (*e.g.* B3LYP, cam-b3lyp, wb97xd, pbe1pbe, mpw1pw91, mn15, m062x, hse1pbe, b3pw91) and the results showed that calculations at MN15 results were the strong agreement with experimental data. This consistency reinforced the reliability of the chosen level of theory for the study.

### 2.2. Calculation of binding constants and detection limits

The binding constants were determined using the Benesi–Hildebrand method (eqn (1)), where *I*_0_ is the absorption or emission intensity of the compound in the absence of the analyte, *I* is the intensity in its presence, and *I*_max_ is the plateau intensity after titration completion:1
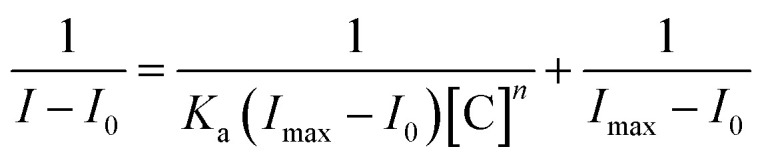


The limit of detection (LOD) was calculated using the following eqn (2):2
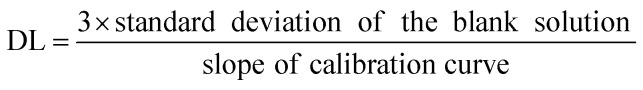


### 2.3. Job's plot analysis

In order to ascertain the stoichiometry of complex, a series of solutions containing HQHBI and DCP with varying mole fractions (*X*) of DCP ranging from 0.1 to 1.0 was prepared where absorption and emission spectra were recorded. Further, according to Job's plot method, a plot of the intensity against the molar fraction of the DCP solution was examined. The complexation ratios of the DCP and HQHBI were calculated using Job's plot.

### 2.4. Synthesis of compound 1a (ref. 32)

8-Hydroxy-2-methyl-quinoline (500 mg, 3 mmol) was added to selenium oxide (1 gm, 9 mmol) in dioxane (20 mL) under inert atmosphere of N_2_ gas. The mixture was heated at 90 °C over 21 hours. On completion, it was filtered and the crude product was purified through column chromatography to obtain compound 1a which was then a yellow solid in a yield of 55%. m.p.: 92–97 °C. ^1^H NMR (CDCl_3_, 400 MHz): *δ* (ppm) 9.93 (s, 1H, –CHO), 8.34 (d, 1H, *J* = 8 Hz), 7.33–7.27 (m, 3H), 6.97–6.93 (m, 1H); ^13^C NMR (DMSO-*d*_6_, 100 MHz): *δ* (ppm) 193.2, 159.4, 152.2, 139.1, 137.6, 136.4, 128.9, 125.8, 120.9, 115.7.

### 2.5. Synthesis of **HQHBI**

**Scheme 1:**
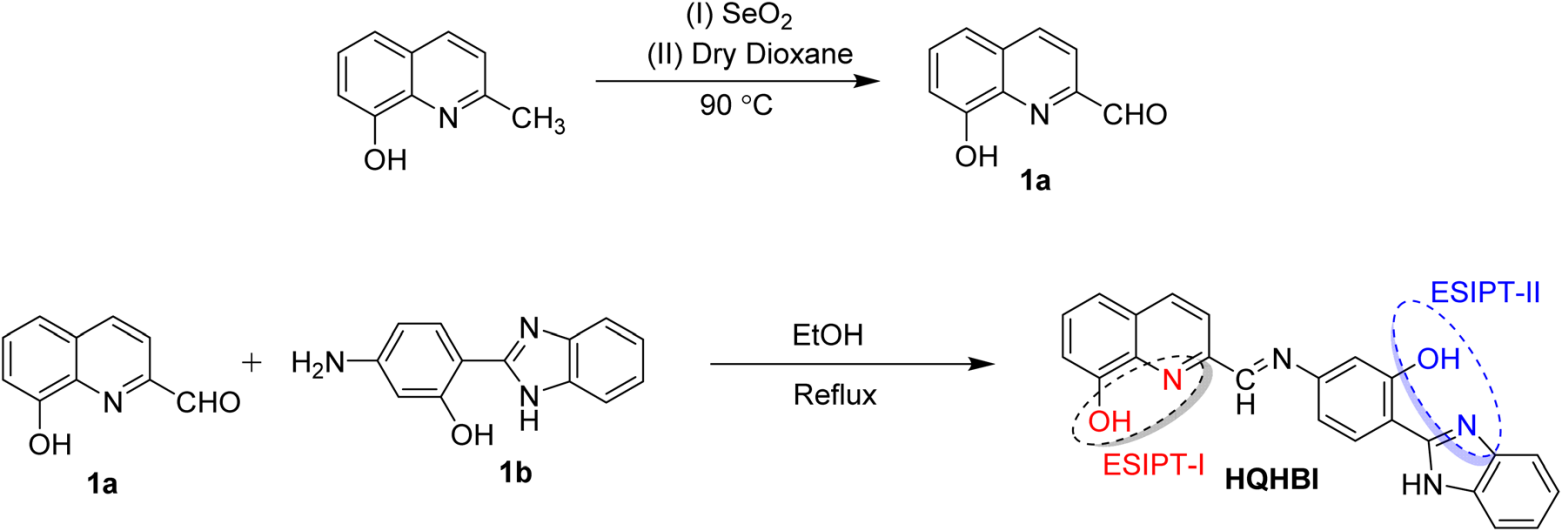
Synthesis of HQHBI.

Compounds 1a and substituted benzimidazole (**1b**)^33^ were refluxed in ethanol for 6 h (Scheme 1). Once the reaction was complete, the mixture was left to cool to room temperature, and the precipitate was obtained by filtration, washing with ethanol and cleaning to yield an orange precipitate (**HQHBI**) in 73% yield. m.p. 250–260 °C. ^1^H NMR (DMSO-*d*_6_, 400 MHz): *δ* (ppm) 8.72 (s, 1H, –C=N), 8.45 (dd, 1H, ^2^*J* = 8 Hz, ^3^*J* = 4 Hz, ArH), 8.00 (d, 1H, *J* = 4 Hz, ArH), 7.87–7.85 (m, 2H, ArH), 7.69 (d, 1H, *J* = 4 Hz, ArH), 7.51–7.45 (m, 4H, ArH), 7.33–7.28 (m, 2H, ArH), 7.06 (dd, 1H, *J* = 4 Hz, ArH) (Fig. S1). ^13^C NMR (DMSO-*d*_6_, 100 MHz): *δ* (ppm) 148.2, 143.8, 142.6, 141.2, 138.8, 138.6, 125.7, 125.5, 124.7, 121.7, 119.7, 119.1, 115.8, 112.5, 108.4, 104.9, 104.1, 103.7, 101.3, 98.6 (Fig. S2). HRMS (ESI-TOF): (*m*/*z*) [M]^+^ calcd for C_23_H_16_N_4_O_2_: 380.1413, found: 380.0142 (Fig. S3).

## 3. Results and discussion

### 3.1. Photophysical properties of **HQHBI**

**Fig. 1:**
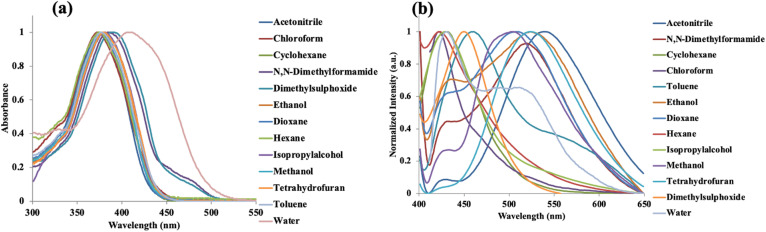
Normalized (a) absorption and (b) fluorescence emission spectra of HQHBI recorded in solvents of different polarity.

The photophysical behavior of HQHBI was systematically determined using both UV-vis absorption and fluorescence spectroscopic technique. In a solution containing 20 µM of HQHBI in CH_3_CN, the resulting absorption spectrum was found to show a strong band with a maximum absorption band at 370 nm. Excitation at this wavelength resulted in a relatively low intensity of fluorescence emission with a maximum of 535 nm, which gave a large Stokes shift of 165 nm. The presence of hydrogen donor and acceptor sites within the HQHBI structure is likely responsible for facilitating both ESIPT and ICT processes. We examined the absorption and emission spectra in various polarity medium to gain understanding into the above-mentioned mechanisms. The absorption maximum for HQHBI in non-polar solvents like hexane and cyclohexane was observed at 370 nm. As the polarity of the solvent was increased, the absorption maximum showed red shift from 370 nm to 380 nm whereas in H_2_O, HQHBI showed absorption maximum at 410 nm. The progressive red shift in the absorption spectra with increasing solvent polarity is ascribed to strengthened ICT in **HQHBI** (Fig. 1a). In non-polar solvents, HQHBI exhibited emission maxima at 420 and 425 nm; however, as the solvent polarity increased, these emission bands shifted to longer wavelengths, appearing at 525 and 535 nm, respectively (Table 1). The observed red shift of 110–115 nm arises from the intramolecular charge transfer (ICT) phenomenon within the molecule. Further, in H_2_O, the emission maximum showed red shift to 428 nm which can be the result of protic solvent interaction with basic imine nitrogen (Fig. 1b). Only a modest red shift of the absorption maximum is observed upon increasing solvent polarity indicating that the transition involves small change in dipole moment whereas, the emission band shows a stronger bathochromic shift which is consistent with fluorescence arising from relaxed ESIPT/ICT state with a larger excited-state dipole, and is strongly stabilized in polar and protic media.

**Table 1:** Photophysical properties of **HQHBI**^a^

S. no.	Solvent	*λ*_max_ (nm)	Molar absorptivity *ε* (M^−1^ cm^−1^)	*λ*_em_ (nm)	Stokes shift Δ*ν* (cm^−1^)	Quantum yield (*Φ*)
1	Hexane	370	7650	420	3200	0.35
2	Cyclohexane	370	7900	425	3500	0.33
3	Toluene	370	12 600	455	5030	0.39
4	Chloroform	370	13 350	420	3200	0.41
5	Tetrahydrofuran	370	11 850	425	3500	0.40
6	Acetonitrile	370	14 600	535	8310	0.44
7	Dioxane	380	15 600	510	7400	0.50
8	Dimethylsulphoxide	380	14 500	515	6900	0.55
9	Dimethylformamide	380	13 500	525	7300	0.51
10	Isopropylalcohol	380	14 650	525	7300	0.57
11	Ethanol	380	14 150	525	7300	0.54
12	Methanol	380	13 900	500	6300	0.52
13	Water	410	9850	428	1100	0.51

a Stokes shift = 1/(*λ*_em_ − *λ*_max_), reference for quantum yield = quinine sulphate.

The observation of dual emission maxima in non-polar solvents that progressively red-shift and merge into a single band in polar media, provides direct experimental evidence for dual-ESIPT activity, with the short-wavelength band arising from the 8-HQ ESIPT channel and the longer one from the benzimidazole site, modulated by solvent polarity effects on H-bonding strength. This assignment is further corroborated by the pH titration data (Fig. 5), where fluorescence enhancement below pH 6 results from protonation of the acceptor nitrogens, inhibiting both ESIPT channels and confirming their proton-dependent nature. The electronic spectra calculated at MN15 functional was close to experimental absorption spectra of synthesized compound and therefore, used for all calculations (Fig. S4).

### 3.2. Spectral characteristics of **HQHBI** towards anions

**Fig. 2:**
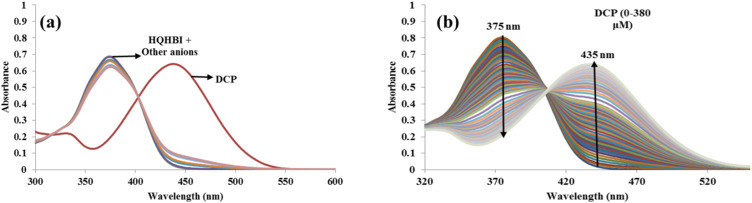
UV-visible absorption spectra of HQHBI (20 µM) in H_2_O : CH_3_CN (1 : 1, v/v, pH 7.1): (a) recorded in the presence of various anions (1000 µM), and (b) obtained upon gradual addition of DCP in the concentration range of 0–380 µM.

The sensing properties of HQHBI were performed in many solvents like CH_3_CN, CH_3_OH, H_2_O as well as CH_3_CN/H_2_O and CH_3_OH/H_2_O mixtures but maximum selectivity and sensitivity were observed in the case of H_2_O : CH_3_CN (1 : 1, v/v, pH = 7.1) solvent. So, preliminary study was accomplished in solution phase H_2_O : CH_3_CN (1 : 1, v/v, pH = 7.1) solvent. The absorption and emission spectra in H_2_O : CH_3_CN (1 : 1, v/v, pH = 7.1) following addition of various anions at 20 µM concentration were monitored for the anion recognition. HQHBI revealed strong absorption maximum at 375 nm in H_2_O : CH_3_CN (1 : 1, v/v, pH = 7.1). Among different anions tested, HQHBI displayed selectivity only for diethyl chlorophosphate (DCP) in H_2_O : CH_3_CN (1 : 1, v/v, pH = 7.1) at 20 µM. Only DCP induced a bathochromic shift, moving the absorption band to 435 nm upon interaction with HQHBI, while no significant spectral changes were observed with other anions including F^−^, Cl^−^, Br^−^, I^−^, HSO_4_^−^, P_2_O_7_^4−^, ClO^−^, H_2_PO_4_^−^, CN^−^, SCN^−^, NO_3_^−^, AcO^−^, DCP, triethylphosphate (TEP), or tributylphosphate (TBP) (Fig. 2a). With increasing concentrations of DCP up to 380 µM, the absorption band at 435 nm showed a gradual enhancement accompanied by a bathochromic shift, while the band centered at 375 nm exhibited a corresponding decrease in intensity (Fig. 2b).

**Fig. 3:**
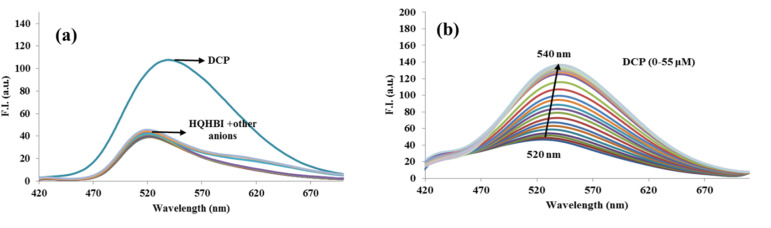
Fluorescence emission spectra of HQHBI (20 µM) in H_2_O : CH_3_CN (1 : 1, v/v, pH 7.1): (a) recorded in the presence of various anions (1000 µM), and (b) obtained upon gradual addition of DCP in the concentration range of 0–55 µM.

The fluorescence spectrum of HQHBI in H_2_O : CH_3_CN (1 : 1, v/v, pH = 7.1) at 20 µM concentration was recorded to observe the fluorescence properties of HQHBI towards DCP. Upon excitation at 370 nm, HQHBI exhibited a maximum emission at 520 nm. Among various anions, HQHBI exhibited selectivity solely for DCP (Fig. 3a). Interaction with DCP enhanced the emission intensity of HQHBI at 540 nm, while other anions induced no significant change in emission intensity. After interacting with DCP, HQHBI formed more widespread π-conjugation systems, resulting in an increase in emission intensity. After the addition of 55 µM of DCP, a plateau in the fluorescence spectra was achieved (Fig. 3b). In the absence of anions, changes in solvent polarity induced only gradual shifts of the spectral positions, reflecting continuous stabilization of the ESIPT/ICT state. On the other hand, addition of DCP to HQHBI in H_2_O : CH_3_CN (1 : 1, v/v, pH = 7.1), generated a new absorption band at 435 nm and a strong fluorescence enhancement at 540 nm, indicative of formation of a distinct state with modified intramolecular hydrogen bonding and conjugation.

**Fig. 4:**
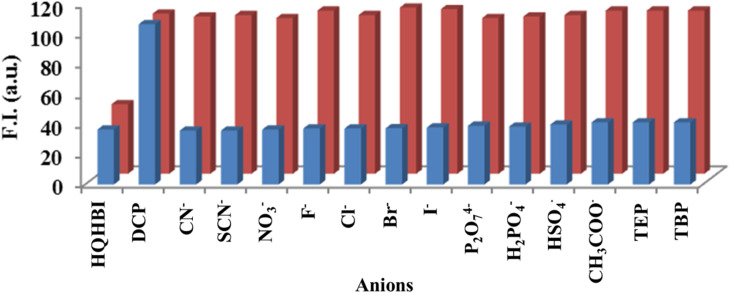
Relative fluorescence emission intensity of HQHBI (20 µM) in H_2_O : CH_3_CN (1 : 1, v/v) at pH 7.1 (*λ*_ex_ = 370 nm) in the presence of various competing anions, recorded at *λ*_em_ = 540 nm. The blue bars denote the emission response of HQHBI with individual anions (1000 µM), while the red bars represent the emission intensity of HQHBI in the presence of DCP along with the respective competing anions (1000 µM).

A competitive experiment confirmed the selective detection of DCP by HQHBI even in the presence of other anions. HQHBI was treated with various anions in the presence of DCP, revealing no significant alteration in fluorescence intensity (Fig. 4). These findings demonstrated that coexisting anions did not interfere with HQHBI's selectivity for DCP.

Fluorescence titration experiments were used to analyze the sensitivity of HQHBI to DCP. Based on the calibration curve, LOD was found to be 1.5 × 10^−7^ M (Fig. S5). The binding affinity was estimated using the Benesi–Hildebrand method by plotting 1/*I*_0_ − *I* against 1/[DCP], which fit a good linear regression (*R*^2^ = 0.9925) and gave a binding constant of 1.5 × 10^4^ M^−1^ (Fig. S6), which represents a 1 : 1 interaction. This stoichiometry was also confirmed by plot analysis of Job which presented highest fluorescence response at a mole fraction of 0.5 (Fig. S7).

### 3.3. Effect of pH on **HQHBI**

**Fig. 5:**
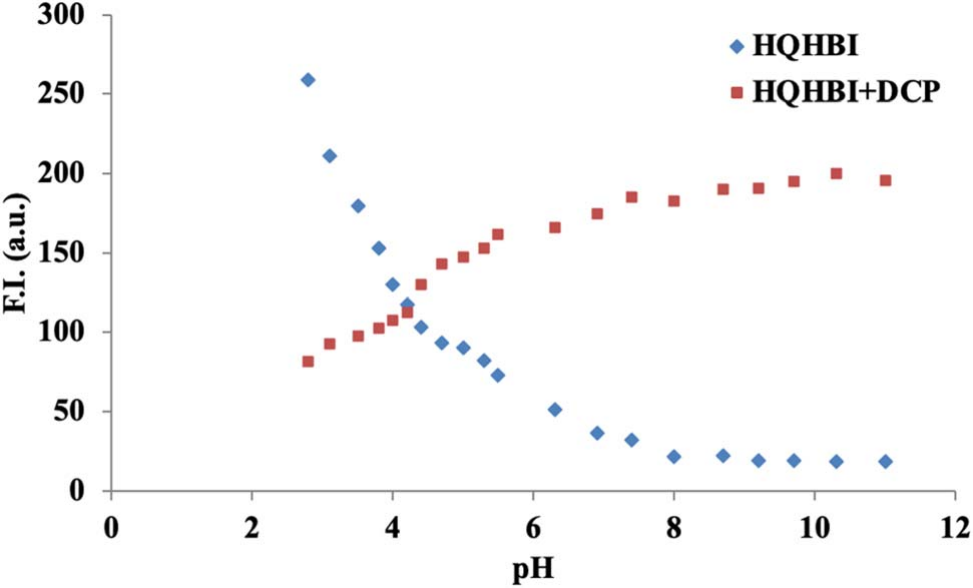
Effect of pH on HQHBI in H_2_O : CH_3_CN, 1 : 1 [v/v] at *λ*_em_ = 540 nm.

pH titrations of HQHBI and it's complex with DCP ion were carried out to examine the practical applicability in a wide pH range. The imine (–C=N–) and hydroxyl (–OH) groups in **HQHBI** facilitated protonation or deprotonation under acidic or basic conditions. In H_2_O : CH_3_CN (1 : 1, v/v), the effect of pH on HQHBI was observed. The HQHBI was stable in the pH range of 6–11 (Fig. 5). The fluorescence intensity in this pH range showed negligible change. Fluorescence intensity at 520 nm increased below pH 6 due to protonation of the hydroxyl oxygen and imine nitrogen atoms, which inhibited the ESIPT process. The HQHBI–DCP complex remained stable across pH 5–11. Thus, DCP detection using HQHBI performed reliably within this pH range.

### 3.4. Time-correlated single photon counting (TCSPC) study

**Fig. 6:**
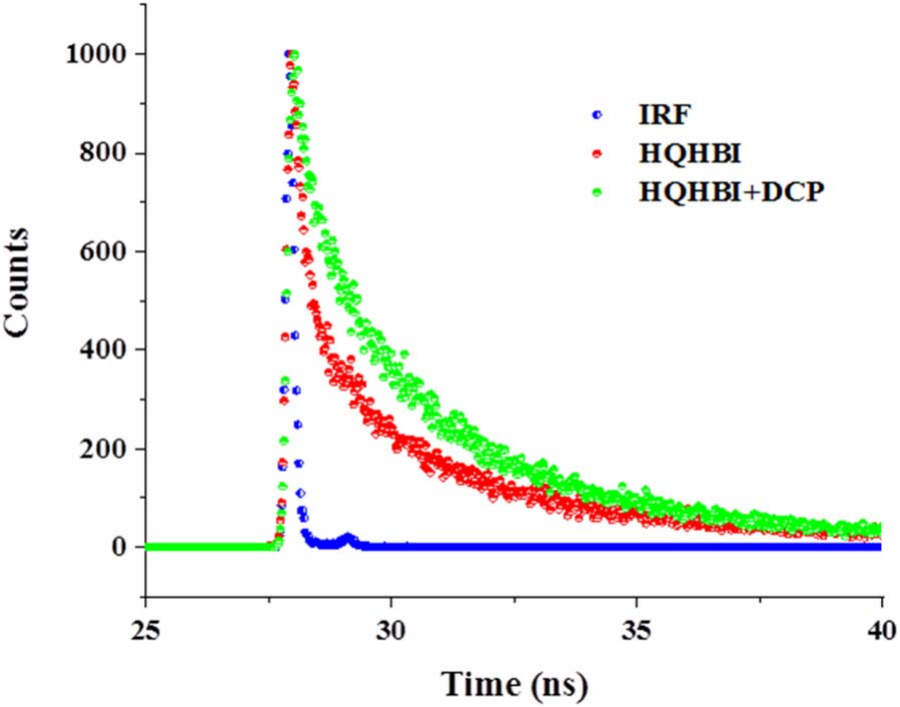
Time resolved fluorescence decay of HQHBI and its complex with DCP at *λ*_em_ = 540 nm in H_2_O : CH_3_CN (20 µM, 1 : 1, [v/v], pH = 7.1).

Fluorescence lifetime measurements performed using time-correlated single photon counting (TCSPC) revealed a noticeable enhancement in the emission lifetime of HQHBI upon interaction with DCP (Fig. 6). The decay curves of free HQHBI as well as its DCP-bound form were adequately described by a tri-exponential fitting model. For HQHBI, three lifetime components of 1.65, 6.26, and 0.15 ns were obtained, with corresponding population contributions of 32.03%, 56.38%, and 11.58%, respectively, resulting in an average lifetime of 0.97 ns. After the addition of DCP, the decay profile exhibited three lifetime components of 2.02, 5.88, and 0.22 ns, contributing 42.36%, 51.19%, and 6.46%, respectively. The average fluorescence lifetime increased to 1.69 ns, as summarized in Table 2. The increase in average lifetime supported the fluorescence enhancement of HQHBI after binding with DCP.

**Table 2:** Fluorescence lifetime measurements for HQHBI and complex with DCP in H_2_O : CH_3_CN (1 : 1, [v/v], 20 µM)

H_2_O : CH_3_CN (1 : 1, [v/v])	*τ*_1_ (ns)	*τ*_2_ (ns)	*τ*_3_ (ns)	*α*_1_	*α*_2_	*α*_3_	*χ* ^2^	*τ*_av_ (ns)
HQHBI	1.65	6.26	0.15	32.03	56.38	11.58	1.08	0.97
**HQHBI** + DCP	2.02	5.88	0.22	42.36	51.19	6.46	1.07	1.69

### 3.5. Plausible sensing mechanism

**Fig. 7:**
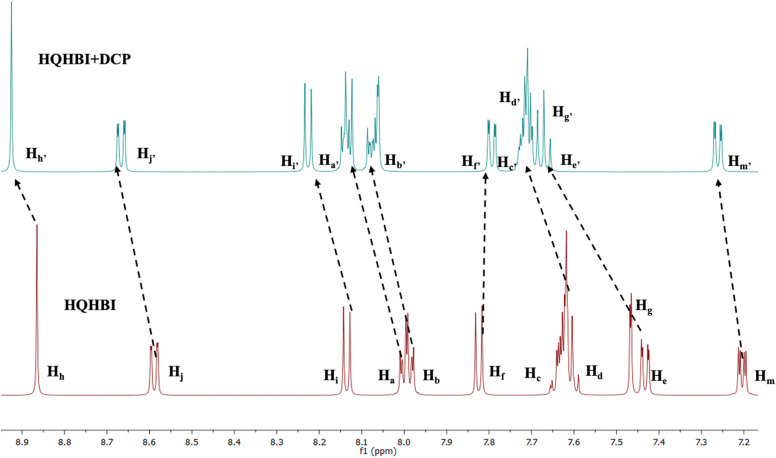
^1^H NMR spectra of HQHBI (5 × 10^−3^ M) upon addition of DCP in CD_3_CN-*d*_3_.

**Scheme 2:**
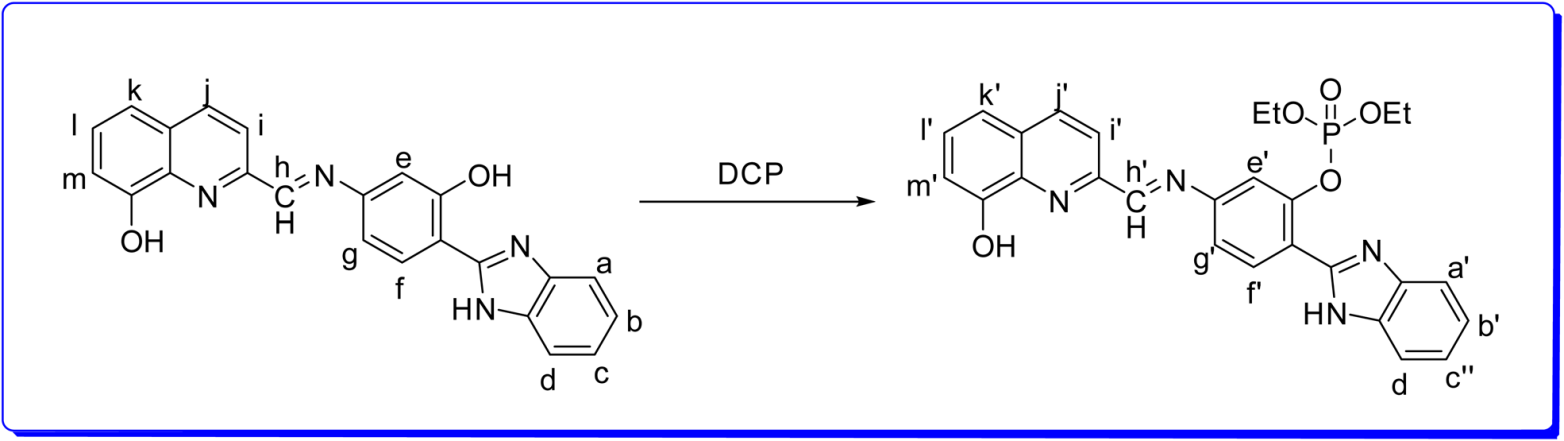
Plausible sensing mechanism for HQHBI and DCP binding.

To find the binding mechanism of HQHBI towards DCP, ^1^H NMR titrations were carried out in CD_3_CN-*d*_3_ (Fig. 7). The singlet at 8.86 ppm corresponds to H_h_ proton due to imine proton of **HQHBI**, experienced a slight downfield shift to 8.93 ppm. The double doublet at 8.59 ppm and 8.14 ppm owing to H_j_ and H_i_ protons of 8-hydroxyquinoline shifted downfield to 8.76 ppm and 8.23 ppm, respectively. The protons at 8.00 ppm due to H_a_ and H_b_ protons of benzimidazole ring were shifted downfield to 8.14 ppm and 8.04 ppm. Further, protons at 7.47 ppm and 7.44 ppm due to H_e_ and H_g_ protons of phenyl ring shifted to 7.69 ppm and 7.67 ppm, respectively. The proton at 7.23 ppm due to H_m_ proton was also slightly shifted downfield. The protons of 8-hydroxyquinoline ring were not affected much which indicated that there is no change in the electronic configuration in 8-hydroxyquinoline ring after the addition of DCP. These results showed that DCP did not bind with –OH group of 8-hydroxyquinoline ring. The changes in ^1^H NMR spectra suggested the binding of DCP through OH group of the phenyl ring (Scheme 2).

### 3.6. Computational studies

**Fig. 8:**
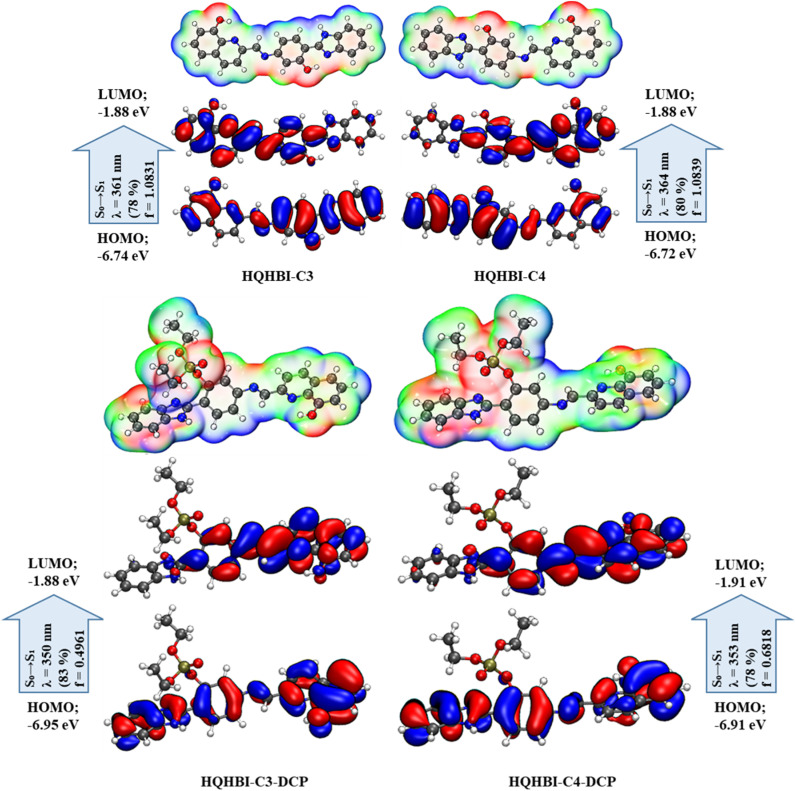
Optimized structures of HQHBI and HQHBI.DCP along with their frontier molecular orbitals.

The ground-state optimized geometries of HQHBI and its complex, HQHBI.DCP, are shown in Fig. 8. In addition, six low-lying Frank–Condon excitations (FCEs) corresponding to the S_0_ geometry of the two conformers of HQHBI were calculated, and three of them are summarized in Table 3. TD-DFT calculations suggest that the S_0_ → S_1_ excitation has a wavelength of 331 nm due to combination of HOMO → LUMO transitions (∼65%) and HOMO → LUMO+1 transitions (∼9%), with the oscillator strength of 1.0578. The calculated result presents a near agreement with the experimentally found absorption maximum (∼370 nm), as summarized in Table 3.

**Table 3:** Summary of excitation spectra of HQHBI and **HQHBI**-DCP

Compound	Transition	Absorption peak (*λ*; nm)	Oscillation strength (f)	Contributing molecular orbitals (MOs, %)
HQHBI	S_0_ → S_1_	331.10	1.0578	H → L (65.6), H → L+1 (9.4), H−5 → L (6.9), H−1 → L (6.5)
	S_0_ → S_2_	286.11	0.0691	H−1 → L (53.7), H−4 → L (13.9), H → L+1 (8.7)
	S_0_ → S_3_	284.08	0.2897	H → L+1 (23.2), H−5 → L (20.8), H−3 → L (12.6), H → L+2 (10.2), H−4 → L (5.4)
**HQHBI**-DCP	S_0_ → S_1_	321.22	0.8006	H → L (61.0), H−1 → L (7.8), H−4 → L (7.1)
	S_0_ → S_2_	286.50	0.1316	H−3 → L (31.2), H−1 → L (20.5), H−2 → L (17.9)
	S_0_ → S_3_	267.58	0.5959	H−4 → L (38.6), H → L+1 (14.2), H−6 → L (7.1), H−5 → L (5.9)

Analysis of the involved molecular orbitals showed the presence of a clear transfer of electron density from benzimidazole (HOMO) to hydroxyquinoline moiety (LUMO+1). Additionally, hole–electron analysis for the S_0_ → S_1_ transition indicates a low spatial overlap between hole and electron distributions (*S*_r_ = 0.6262) and a substantial separation between their centroids (*D* = 1.355 Å), providing strong evidence for an intramolecular charge transfer (ICT) process in **HQHBI** (Fig. 8).

Furthermore, the S_0_ to S_2_ and S_0_ to S_3_ transitions were estimated at wavelengths of 286 nm and 284 nm, respectively, of oscillator strengths of 0.0691 and 0.2897. In addition, the molecular electrostatic potential (MEP) of HQHBI was analyzed to assess its relative reactivity towards nucleophilic and electrophilic interactions. The negative potential regions (depicted in red and yellow) and the positive potential regions (depicted in blue) in MEP represent the electrophilic and nucleophilic reactivity positions, respectively, as shown in Fig. 8. The most significant negative electrostatic potential is observed at the oxygen atoms which found in the phenolic unit and quinoline unit. The positive regions, which are sites of nucleophilic reactivity, found at the hydrogen atoms attached to the periphery of HQHBI.

The MEP analysis also reveals that the DCP analyte can form electrophilic interactions with the phenolic moiety's hydroxyl group. Complex HQHBI.DCP was thus optimized, and electronic spectra of HQHBI when interacting with DCP were re-calculated. Calculations show that the S_0_ → S_1_ transition of HQHBI.DCP at 321 nm is dominated by the overlap of HOMO → LUMO (∼61%) transition with an oscillator strength of 0.8006. The molecular orbitals involved in this transition have a redistribution of the electron density from the benzimidazole moiety (HOMO) to the quinoline moiety (LUMO). Hole–electron analysis of compound HQHBI.DCP shows a smaller overlap integral (*S*_r_ = 0.62081) and greater separation of electron and hole centroids (*D* = 1.303 Å), which signifies a smaller intramolecular charge transfer process in HQHBI.DCP. S_0_ → S_2_ and S_0_ → S_3_ transitions were also computed in HQHBI.DCP at 286 nm and 267 nm, respectively, with corresponding oscillator strengths of 0.1316 and 0.5959. The calculated red shift of the S_0_ → S_1_ transition from 331 nm (HQHBI) to 321 nm (HQHBI–DCP) reflects a smaller HOMO–LUMO gap in the complex, arising from DCP-induced stabilization of the LUMO (quinoline-localized) while weakly raising the HOMO (benzimidazole/phenyl), thereby enhancing ICT character and lowering the excitation energy. This theoretical red shift aligns with the experimental bathochromic shift observed in absorption (375 → 435 nm), confirming that binding facilitates more efficient charge delocalization rather than contradicting the turn-on sensing mechanism.

The ^1^H NMR downfield shifts of phenyl protons confirmed DCP binding disrupts phenolic H-bonding, which TD-DFT analysis correlated with reduced HOMO–LUMO spatial overlap. The TCSPC lifetime increase reflects radiative decay consistent with the calculated lower S_1_ oscillator strength favoring emissive channels. This multi-method correlation establishes that DCP coordination activates ESIPT while suppressing non-radiative ICT quenching.

### 3.7. Application in molecular keypad lock

The interaction of HQHBI with DCP enables the stimulation and construction of Boolean logic gates capable of performing molecular-level mathematical operations in a comprehensive manner. The development of molecular logic gates is based on chemical inputs obtained from spectroscopic data.

**Fig. 9:**
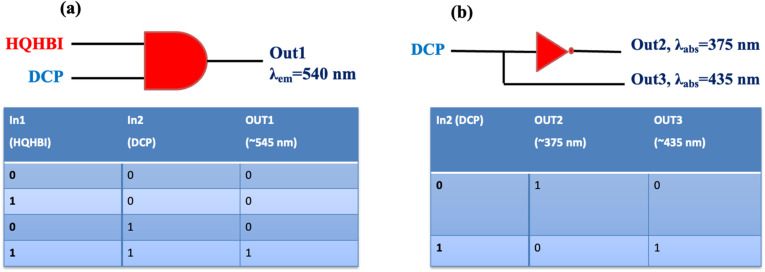
(a) Fluorescence emission response upon the addition of In1 = HQHBI and In2 = DCP, along with the corresponding truth table and its 2-to-1 encoder representation; (b) UV-visible absorption response upon the addition of In1 = DCP, accompanied by the associated truth table and its 1-to-2 decoder representation.

According to the results of fluorescence spectroscopy, HQHBI can simulate a wide range of logic gate assembly using two chemical inputs: HQHBI (In1) and DCP (In2) (Fig. 9). There was just one output which represented the emission peak of HQHBI at 540 nm. The 2-to-1 combination logic encoder is intended to have a single output and dual inputs. A “0” value has been allocated to the “off” state for emission intensity less than threshold value, while “1” value has been assigned to the “on” state for emission intensity greater than threshold value. The output is “on” only in the case when both the inputs are present. The AND gate was created as a result of the data combination.

A 1-to-2 decoder based on a single input was constructed, where In1 corresponds to DCP, Out2 represents the absorption maximum at 375 nm, and Out3 corresponds to the absorption maximum at 435 nm. In the presence of the input, Out3 switches to the “on” state while Out2 remains “off,” whereas in the absence of the input, Out2 is “on” and Out3 is “off.” These observations satisfy the required conditions for the realization of a 1-to-2 decoder.

## 4. Conclusion

In summary, 8-hydroxyquinoline derivative (HQHBI) has been developed as a highly sensitive and selective fluorescent chemosensor for the detection of the organophosphate simulant diethyl chlorophosphate (DCP) (Table S1). The probe exhibits excellent photophysical properties, including a large Stokes shift, solvent-dependent ESIPT/ICT behavior, and enhanced fluorescence upon interaction with DCP. Its remarkable selectivity in the presence of competing anions underscores its practical applicability in organophosphate sensing. With a low detection limit of 1.5 × 10^−7^ M, HQHBI is capable of detecting trace levels of DCP relevant to environmental monitoring and security applications. Mechanistic insights obtained from NMR titration, pH studies, time-resolved fluorescence, and computational (DFT/TD-DFT) analyses confirm selective recognition *via* the phenolic –OH group, leading to electronic reorganization and fluorescence modulation. Furthermore, the ability of HQHBI to function as a molecular keypad lock and perform logic gate operations highlights its potential in molecular computing. Overall, HQHBI represents a promising platform for next-generation DCP detection, combining sensitivity, selectivity, structural tunability, and multifunctional photophysical properties.

## Author contributions

Aastha Palta: writing – original draft, methodology, investigation, formal analysis. Gulshan Kumar: computational studies. Kamaldeep Paul: supervision, validation, funding acquisition, Vijay Luxami: review, editing, supervision, resources, funding acquisition.

## Conflicts of interest

The authors affirm that they do not have any financial or personal affiliations that might have biased the research reported in this manuscript.

## Supplementary Material

RA-016-D6RA01529H-s001

## Data Availability

The data supporting this article have been included as part of the supplementary information (SI). Supplementary information (SI) is available. See DOI: https://doi.org/10.1039/d6ra01529h.
