# Supplementary material for: ESIPT-active 8-hydroxyquinoline chemosensor for highly selective detection of diethyl chlorophosphate and molecular logic gate applications
Source: RSC Adv. 2026 May 11;16(27):24568–78. doi: 10.1039/d6ra01529h (PMC13159071; doi:10.1039/d6ra01529h)
Supplement: RA-016-D6RA01529H-s001 [file RA-016-D6RA01529H-s001.pdf]

Supplementary Information (SI) for RSC Advances.
This journal is © The Royal Society of Chemistry 2026

Supporting Information

ESIPT-Active 8-Hydroxyquinoline Chemosensor for Highly Selective Detection of Diethyl Chlorophosphate and Molecular Logic Gate Applications

Aastha Palta[a], Gulshan Kumar[b], Kamaldeep Paul[c] and Vijay Luxami[c]*

[a]*University Centre for Research and Development, Chandigarh University, Mohali-140413, India*

[b]*Department of Chemistry, Banasthali University, Banasthali Newai, 304022 Rajasthan, India*

[c]*Department of Chemistry and Biochemistry, Thapar Institute of Engineering and Technology, Patiala-147001, India*

Email: vluxami@thapar.edu

Table of Contents:

Figure S1: ^{1}H NMR spectrum of **HQHBI**.

Figure S2: ^{13}C NMR spectrum of **HQHBI**.

Figure S3: HRMS spectrum of **HQHBI**.

Figure S4: Comparison of calculated absorption spectra at different functional with experimental absorption spectra peak.

Figure S5: Plot for the determination of lowest detection limit of **HQHBI** (20 μM) with DCP in H_2O: CH_3CN, 1:1 [v/v].

Figure S6: Benesi-Hildebrand plot for determination of binding constant of **HQHBI** (20 μM) with DCP in H_2O: CH_3CN, 1:1 [v/v].

Figure S7: Job's Plot for **HQHBI** (20 μM) binding with DCP (20 μM) in H_2O: CH_3CN, 1:1 [v/v].

Table S1: Comparison with previously reported DCP sensors.

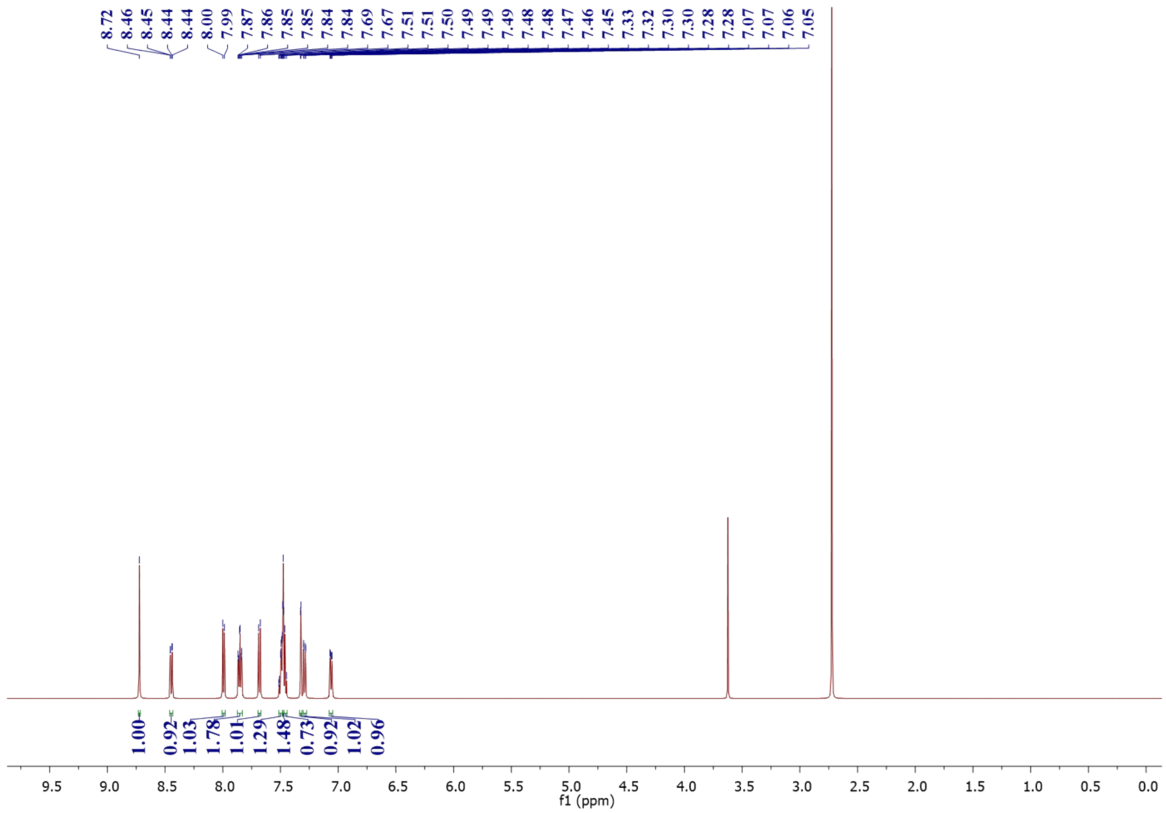

Figure S1: ^{1}H NMR spectrum of **HQHBI**.

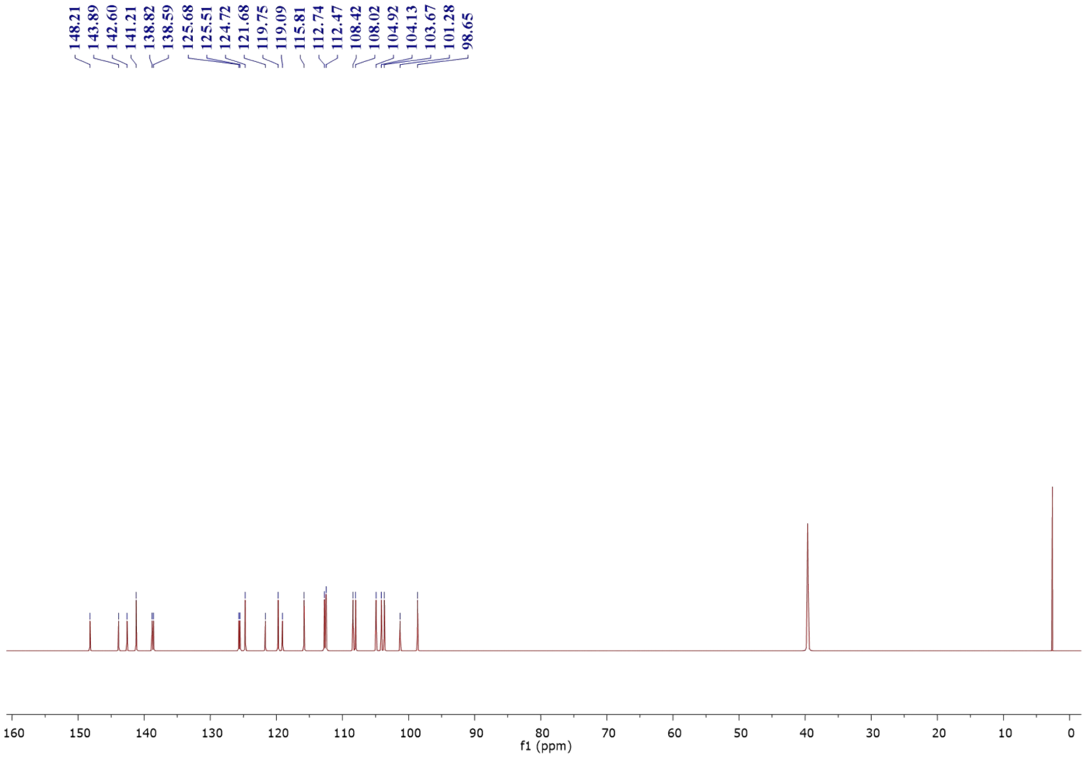

Figure S2: ^{13}C NMR spectrum of **HQHBI**.

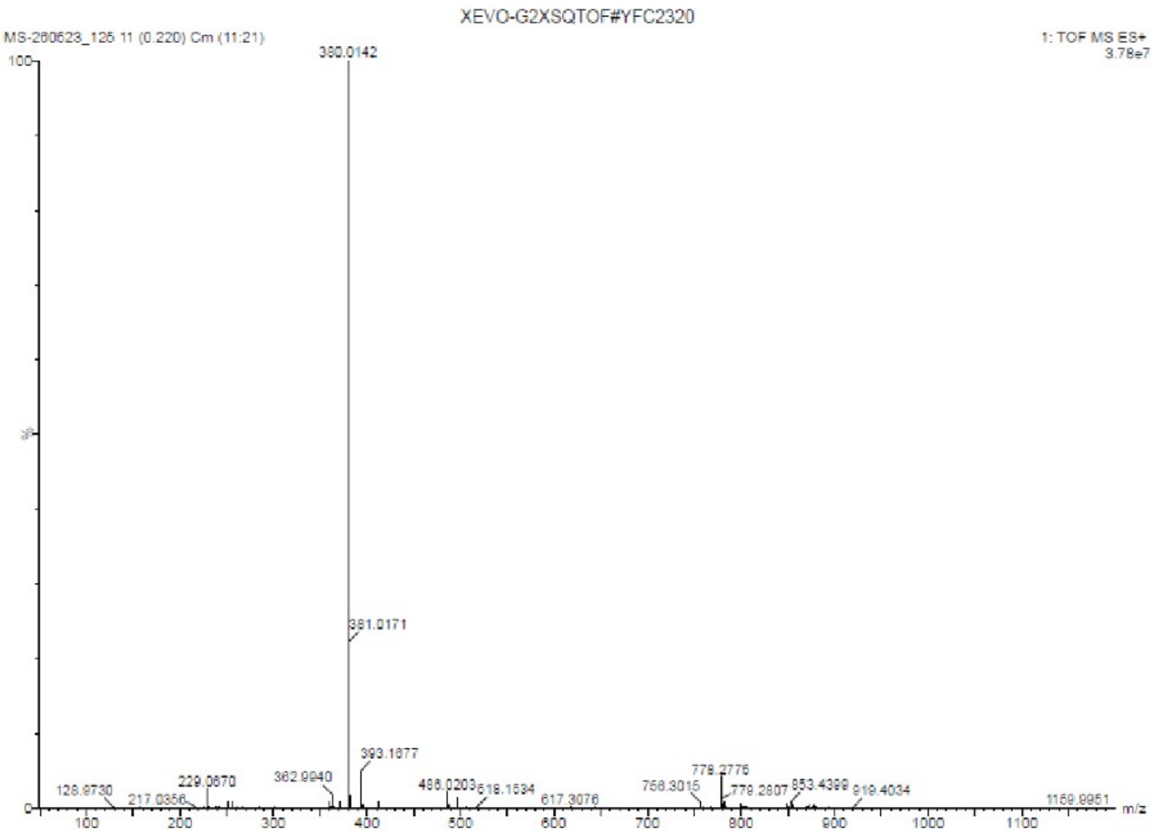

Figure S3: HRMS spectrum of **HQHBI**.

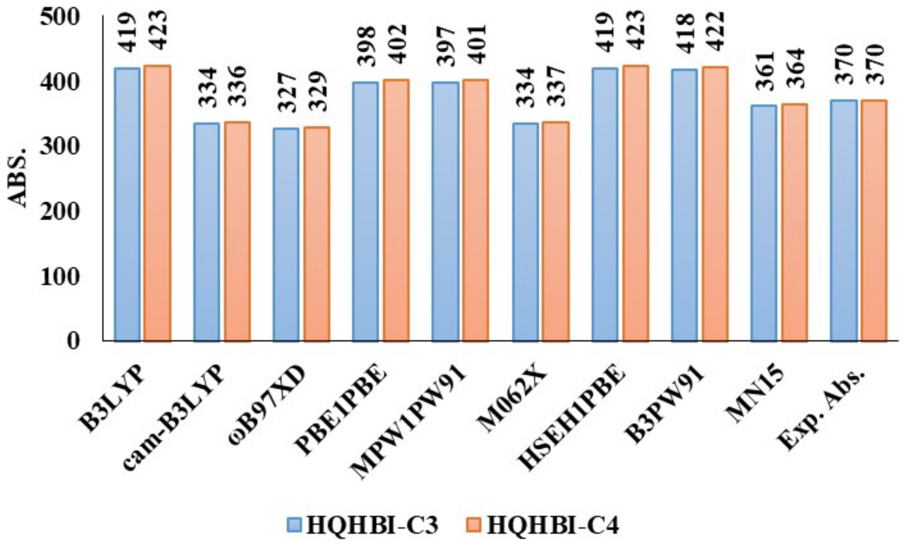

Figure S4: Comparison of calculated absorption spectra at different functional with experimental absorption spectra peak.

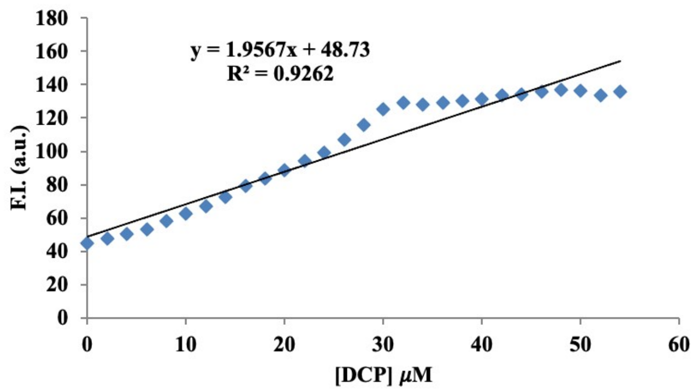

Figure S5: Plot for the determination of lowest detection limit of **HQHBI** (20 μM) with DCP in H_2O: CH_3CN, 1:1 [v/v].

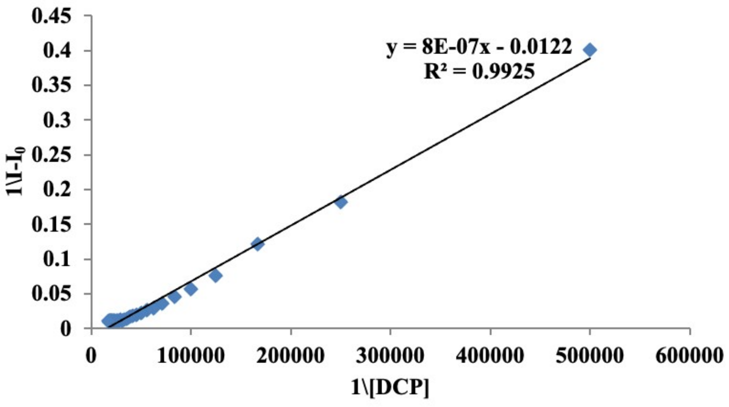

Figure S6: Benesi-Hildebrand plot for determination of binding constant of **HQHBI** (20 μM) with DCP in H_2O: CH_3CN, 1:1 [v/v].

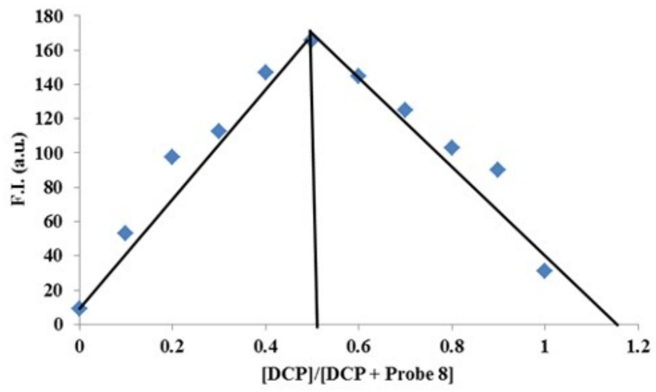

Figure S7: Job's Plot for **HQHBI** (20 μM) binding with DCP (20 μM) in H_2O: CH_3CN, 1:1 [v/v].

Table S1: Comparison with previously reported DCP sensors.

S. No.:	Structure	Solvent	Selectivity	Limit Of detection	References
1	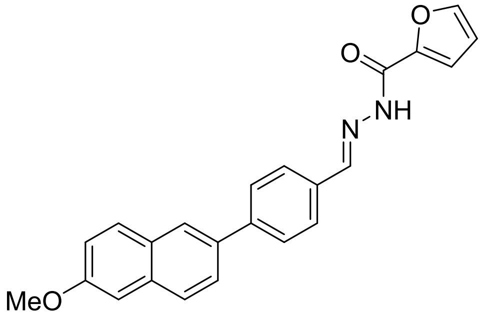	CH_3CN	DCP	12.2 nM	1
2	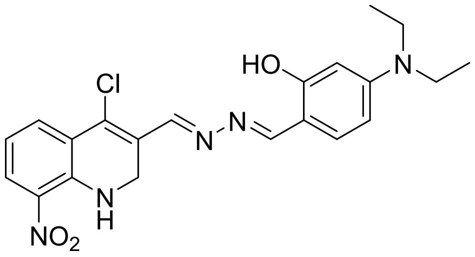	DMF	DCP	1.5µM	2
3	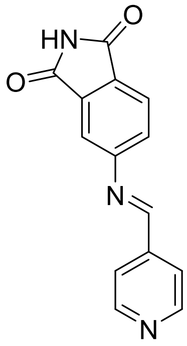	CH_3CN	DCP	24 nM	3
4	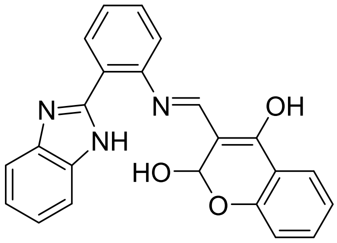	CH_3CN	DCP	6.6 µM	4
5	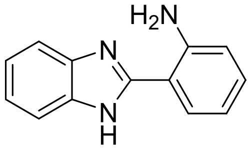	CH_3CN	DCP	20.9 µM	5
6	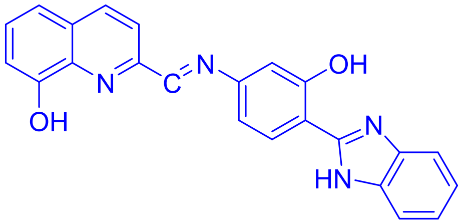	H_2O: CH_3CN, 1:1 [v/v]	DCP	0.15 µM	This Work

1. S. Banerjee, P. Ghosh, A. Karak, D. Banik, and A. K. Mahapatra, A chemodosimetric chemosensor for the ratiometric detection of nerve agent-mimic DCP in solution and vapor phases, *Anal. Methods, 2025,**17**, 432-439.*
2. S.S.Ramasamy, K. Adhigaman, V. Nandakumar, A. Muralidharan, S. Ramasamy, and S. Thangaraj, Design and synthesis of 8-nitroquinoline azine D-π-A module chemosensors: Fluorogenic onsite detection of sarin gas mimic-DCP, *Talanta Open*, 2025, **12**, 100508.
3. X. Wen, T. Jiang, T. Liu, B. Zhao, X. Xu, H. Jeong, C. Fan, Y. Sun, J. Yoon, and Z. Lu, Schiff base-based turn-on fluorescent probe for rapid on-site detection of nerve agent simulants in solution and vapor, *Microchem. J.,* 2026, **224**, 117473.
4. N. Tohora, C. Debnath, S. Ahamed, J. Chourasia, M. Mahato, S. Ali, S. Lama, S. Pradhan, and S. K. Das, An efficient ESIPT-based ratio-/fluorimetric probe for rapid and sensitive detection of the sarin surrogate diethylchlorophosphate in solution and vapor phases, *Anal. Methods*, 2025,**17**, 2067-2075.
5. A. D. Jaiswal, J. Chourasia, S. Ahamed, N. Tohora, S. Lama, M. Mahato, U. Darnal, S. Ghanta, and S. K. Das, Decrypting the mechanistic aspect of a fluorochromic diethylcholorophosphate sensitive benzimidazole based probe: A combined spectroscopic and theoretical investigation, *Microchem. J.,* 2025, **212**, 113421.
